# Strongyloidiasis and Diffuse Alveolar Hemorrhage in a Patient with Systemic Lupus Erythematosus

**DOI:** 10.1155/2014/278390

**Published:** 2014-06-12

**Authors:** Fernando Gonzalez-Ibarra, Parag Chevli, Lindsey Schachter, Maninder Kaur, Sahar Eivaz-Mohammadi, Basheer Tashtoush, Jioty Matta, Amer K. Syed, Valentin Marian

**Affiliations:** ^1^Department of Internal Medicine, Mount Sinai School of Medicine, Jersey City Medical Center, 355 Grand Street, Jersey City, NJ 07302, USA; ^2^Department of Internal Medicine, St. George's University School of Medicine, Jersey City Medical Center, 355 Grand Street, Jersey City, NJ 07302, USA; ^3^Department of Pulmonary and Critical Care Medicine, Cleveland Clinic Florida, Weston, FL 33331, USA; ^4^Department of Pulmonary and Critical care, Mount Sinai School of Medicine, Jersey City Medical Center, 355 Grand Street, Jersey City, NJ 07302, USA; ^5^Laureate National Institute of Medicine, Jersey City Medical Center, 355 Grand Street, Jersey City, NJ 07302, USA; ^6^Department of Rheumatology, Mount Sinai School of Medicine, Jersey City Medical Center, 355 Grand Street, Jersey City, NJ 07302, USA

## Abstract

The presence of *Strongyloides stercoralis* infection in patients with systemic lupus erythematosus (SLE) has been described previously. *Strongyloides stercoralis* hyperinfection syndrome (SHS) that usually develops in patients under immunosuppressive therapy may affect a variety of organs, but the presentation with diffuse alveolar hemorrhage (DAH) is rare with only a few cases described in the literature. We present the case of a 36-year-old Hispanic female with a past medical history relevant for SLE and a recent diagnosis of lupus nephritis and hypertension. The patient who developed sudden and progressive abdominal pain and respiratory distress, with the presence of bilateral crackles and severe hypoxemia, is currently under treatment with steroids and cyclophosphamide for worsening of lupus nephritis. The patient underwent endotracheal intubation and mechanical ventilation, and computed tomography showed the presence of bilateral pulmonary infiltrates suggestive of DAH. Bronchoalveolar lavage was done and showed the presence of filariform larvae, morphologically consistent with *Strongyloides stercoralis*. Treatment with ivermectin was started and patient responded to treatment with improvement of clinical status. In conclusion, the development of SHS in patients with lupus, especially when receiving immunosuppressive therapy, is a severe and potentially fatal complication. Early detection and treatment may decrease mortality.

## 1. Introduction 

Systemic lupus erythematosus is an autoimmune disease that causes potentially life-threatening flares. The disease affects all systems of the body and may be manifested in differing degrees of severity. In 1904, Sir Osler suggested a pulmonary component associated with the disease [1]. These pleuropulmonary manifestations have since been widely reported in the literature, with involvement affecting 50–70% of patients. Lung involvement, although frequent in SLE patients, is often secondary to other organ manifestations or as a side effect of treatments [2].

SLE patients, particularly those experiencing a flare-up, are treated with corticosteroids, which causes an immunocompromised state, putting these individuals at further increased risk for bacterial, fungal, and parasitic infections. One such infection is that of* Strongyloides stercoralis*, which is endemic in tropical and subtropical regions. This parasite completes its asexual life cycle within its human host, with the potential to cause persistent infection leading to reinfection and ultimately what is known as a hyperinfection syndrome (HS). This hyperinfection causes exacerbation of symptoms, particularly those affecting the gastrointestinal and pulmonary systems [3].

The complication of diffuse alveolar hemorrhage (DAH) in patients with SLE may develop over several hours and is often fatal. Mortality rates for SLE patients with DAH have been reported to be as high as 70–90%, even in those with noninfectious cases [4]. While DAH is a rare manifestation in SLE patients, those infected with disseminated strongyloidiasis may be at an increased risk for this severe complication because of the increased stress to the pulmonary system. The literature has few reports describing fatal disseminated strongyloidiasis leading to alveolar hemorrhage. Here we present one such case.

## 2. Case Presentation 

This patient, a 36-year-old Hispanic female, with a past medical history of SLE, hypertension, and lupus nephritis, was admitted to the hospital for a urinary tract infection and worsening of lupus nephritis. The diagnosis of lupus and lupus nephritis was apparently recent and patient was taking prednisone 20 mg PO daily at home, but she did not follow up properly as an outpatient and she was not apparently taking any other medications.

During the hospital admission, patient was started a pulsed dose of methylprednisolone 750 mg IV daily for 3 days and cyclophosphamide. However, ten minutes after the cyclophosphamide administration (500 mg IV infusion), the patient developed a severe allergic reaction, characterized by tachycardia, tachypnea, and hemodynamic instability and was treated with epinephrine 0.2 mL subcutaneously and continued with steroids IV. Minimal response of renal function was observed and during hospital course patient experienced recurrent episodes of diffuse abdominal pain and mild abdominal distention accompanied by progressive hypoxemic respiratory failure.

Physical examination was relevant for tachypnea (20–24 breaths per minute) with obvious use of her accessory muscles of respiration and bilateral diffuse crackles appreciated on auscultation of her lung fields, the patient required respiratory support and was placed on mechanical ventilation.

**Figure 1:**
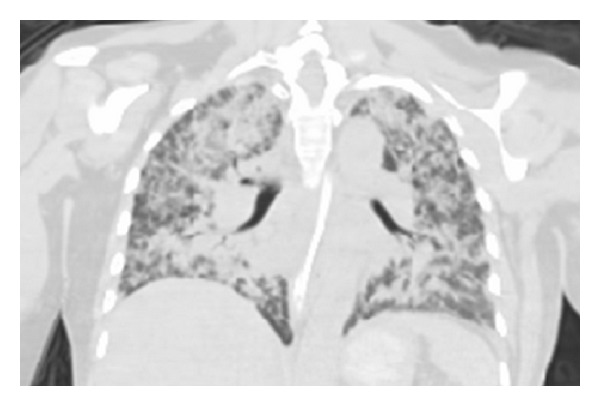
CT thorax demonstrating diffuse airspace opacities involving bilateral lung fields.

**Figure 2:**
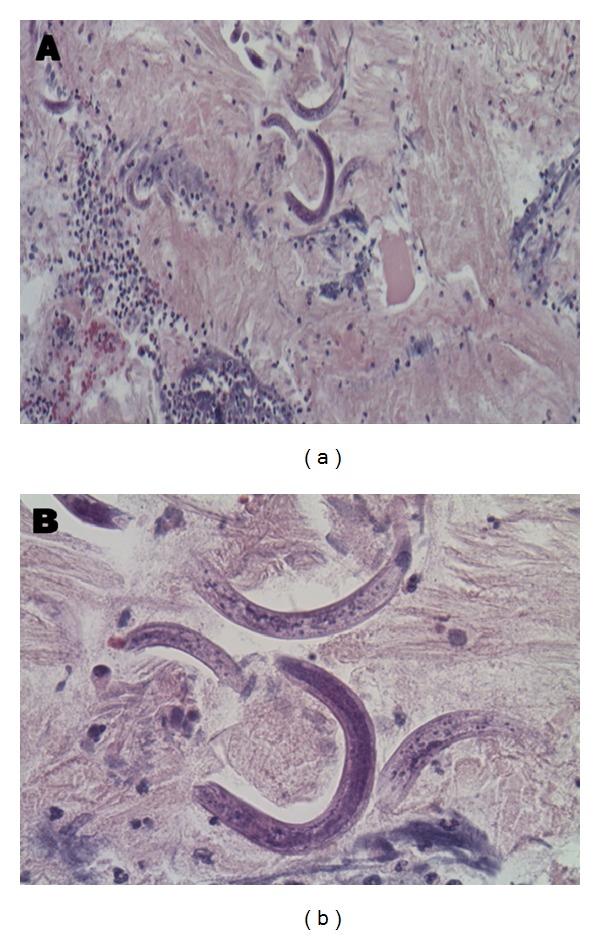
(a) and (b) Pathology shows bronchial epithelial cells, alveolar macrophages, and inflammatory cells but is negative for malignant cells. Filariform larvae, morphologically consistent with* Strongyloides stercoralis*, are present.

**Figure 3:**
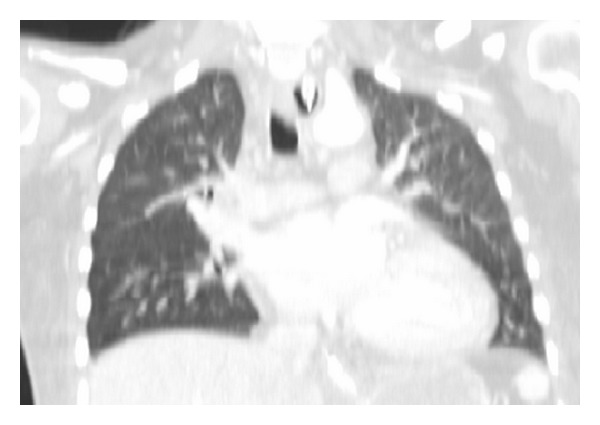
CT thorax four months after treatment demonstrates improvement in airspace opacities.

Computed tomography (CT) of the thorax showed diffuse airspace opacities (Figure 1). Bronchoalveolar lavage (BAL) was performed and it revealed diffuse bilateral pulmonary hemorrhage and filariform larvae, morphologically consistent with* Strongyloides stercoralis* (Figures 2(a) and 2(b)). The patient was treated with ivermectin with a favorable response. CT thorax at four months of follow-up demonstrated an improvement in airspace opacities (Figure 3).

## 3. Discussion and Conclusions

*Strongyloides stercoralis* is a parasite endemic to tropical and subtropical regions. The parasite invades the intact skin of its host, traveling via the lymphatic system to the venous system, which carries the parasite to the lungs. In the lungs, it is able to penetrate through the alveolar spaces. It is then able to travel up the trachea, is swallowed, and reaches the preferred location in the duodenum and jejunum of the small intestine. Within the intestine, it may penetrate the bowel wall, leading to an autoinfection state. In the immunocompromised population, this autoinfection may lead to immense proliferation of the parasite, causing what is known as HS [5].

Although many infected individuals are asymptomatic, once the parasite is in the lungs it may cause chest burning, wheezing, and coughing, along with pneumonia-like symptoms (Loffler's syndrome). The development of HS in and of itself, in patients with SLE, particularly those receiving immunosuppressive therapy, is a severe and potentially fatal complication. With HS affected patients, the risk of DAH increases, increasing fatally risk [6].

As in the above case, there was evidence of pulmonary symptoms and identification of* Strongyloides Stercoralis* in the respiratory tract, with evidence of both pulmonary and gastrointestinal symptoms. It is likely that she had been living with an asymptomatic parasitic infection for a long time. However, once she was in an important immunosuppressed state due to her SLE flare-up, it created an unbalanced condition, where she was unable to keep the parasite suppressed, presenting with pulmonary affection characterized by DAH.

Early detection and treatment may help to decrease mortality in these patients [7]. Therefore, it is essential to investigate for the presence of* Strongyloides Stercoralis* in immunocompromised individuals, with any risk factors, even those currently living in nonendemic areas.
